# Racial and Ethnic Disparities in Rates of Invasive Second Breast Cancer Among Women With Ductal Carcinoma In Situ in Hawaiʻi

**DOI:** 10.1001/jamanetworkopen.2021.28977

**Published:** 2021-10-20

**Authors:** Kekoa Taparra, Jami Fukui, Jeffrey Killeen, Kenneth Sumida, Lenora W. M. Loo, Brenda Y. Hernandez

**Affiliations:** 1 Department of Radiation Oncology, Stanford Health Care, Stanford, California; 2 University of Hawaiʻi Cancer Center, University of Hawaiʻi at Mānoa, Honolulu; 3 Department of Pathology, Kapiʻolani Medical Center for Women and Children, Honolulu, Hawaiʻi; 4 Department of Medicine, University of Hawaiʻi John A. Burns School of Medicine, Honolulu

## Abstract

**Importance:**

Women with ductal carcinoma in situ (DCIS) may develop a subsequent invasive second breast cancer (SBC). Understanding the association of racial and ethnic factors with the development of invasive SBC may help reduce overtreatment and undertreatment of women from minority groups.

**Objective:**

To evaluate risk factors associated with developing invasive ipsilateral SBC (iiSBC) and invasive contralateral SBC (icSBC) among women with an initial diagnosis of DCIS who are from racial and ethnic minority populations.

**Design, Setting, and Participants:**

This retrospective cohort study used deidentified data from the Hawaiʻi Tumor Registry of 6221 female Hawaiʻi residents aged 20 years or older who received a diagnosis of DCIS between January 1, 1973, and December 31, 2017. The 5 most populous ethnic groups were compared (Chinese, Filipino, Japanese, Native Hawaiian, and White). Data analysis was performed from 2020 to 2021.

**Exposures:**

Patient demographic and clinical characteristics and the first course of treatment.

**Main Outcome and Measures:**

The a priori study outcome was the development of invasive SBC. Logistic regression was used to identify factors associated with invasive SBC. Factors that were significant on unadjusted analyses were included in the adjusted models (ie, age, race and ethnicity, diagnosis year, DCIS histologic characteristics, laterality, hormone status, and treatment).

**Results:**

The racial and ethnic distribution of patients with DCIS across the state’s most populous groups were 2270 Japanese women (37%), 1411 White women (23%), 840 Filipino women (14%), 821 Native Hawaiian women (13%), and 491 Chinese women (8%). Women of other minority race and ethnicity collectively comprised 6% of cases (n = 388). A total of 6221 women (age range, 20 to ≥80 years) were included in the study; 4817 (77%) were 50 years of age or older, 4452 (72%) received a diagnosis between 2000 and 2017, 2581 (42%) had well or moderately differentiated histologic characteristics, 2383 (38%) had noninfiltrating intraductal DCIS, and 2011 (32%) were treated with mastectomy only. Of these 6221 women, 444 (7%) developed invasive SBC; 190 developed iiSBC (median time to SBC diagnosis, 7.8 years [range, 0.5-30 years]) and 254 developed icSBC (median time to SBC diagnosis, 5.9 years [range, 0.5-28.8 years]). On adjusted analysis, women who developed iiSBC were more likely to be younger than 50 years (adjusted odds ratio [aOR], 1.49; 95% CI, 1.08-2.06), Native Hawaiian (aOR, 3.28; 95% CI, 2.01-5.35), Filipino (aOR, 1.94; 95% CI, 1.11-3.42), Japanese (aOR, 1.58; 95% CI, 1.01-2.48), and untreated (aOR, 2.29; 95% CI, 1.09-4.80). Compared with breast-conserving surgery (BCS) alone, there was a decreased likelihood of iiSBC among women receiving BCS and radiotherapy (aOR, 0.45; 95% CI, 0.27-0.75), BCS and systemic treatment with or without radiotherapy (aOR, 0.40; 95% CI, 0.23-0.69), mastectomy only (aOR, 0.23; 95% CI, 0.13-0.39), and mastectomy and systemic treatment (aOR, 0.57; 95% CI, 0.33-0.96). Women who developed an icSBC were more likely to be Native Hawaiian (aOR, 1.69; 95% CI, 1.10-2.61) or Filipino (aOR, 1.70; 95% CI, 1.10-2.63). Risk of both iiSBC and icSBC decreased in the later years of diagnosis (2000-2017) compared with the earlier years (1973-1999).

**Conclusions and Relevance:**

This study suggests that Native Hawaiian and Filipino women who initially received a diagnosis of DCIS were more likely to subsequently develop both iiSBC and icSBC. Japanese women and younger women were more likely to develop iiSBC. Subpopulation disaggregation may help guide clinical treatment and screening decisions for at-risk subpopulations.

## Key Points

**Question:**

Is there variation in the risk for developing an invasive second breast cancer (SBC) among women with an initial diagnosis of ductal carcinoma in situ who are from racial and ethnic minority populations?

**Findings:**

This cohort study including 6221 women found that Native Hawaiian and Filipino women were at significantly higher risk than White women of developing both invasive ipsilateral SBC and invasive contralateral SBC.

**Meaning:**

This study suggests that understanding the racial and ethnic differences in the potential risk of invasive SBC using data sets with a more diverse population may help guide clinical treatment and screening decisions for at-risk subpopulations.

## Teaser

This cohort study uses data from the Hawaiʻi Tumor Registry to evaluate risk factors associated with developing invasive ipsilateral and contralateral second breast cancer among women from racial and ethnic minority populations with an initial diagnosis of ductal carcinoma in situ.

## Introduction

Invasive breast cancer is the most common cancer among US women, with more than 280 000 diagnoses and 40 000 deaths expected in 2021.^1^ It remains the leading cause of death for women 20 to 59 years of age, and improvements in mortality have steadily plateaued across age groups.^1^ With improvements in mammography screening, the number of noninvasive ductal carcinoma in situ (DCIS) diagnoses has increased.^2^ Although DCIS outcomes are generally favorable, up to 40% of women develop a second breast cancer (SBC) after DCIS, 28% of which are invasive breast cancers.^3,4^ Optimal therapeutic and clinical follow-up strategies for DCIS remain a topic of discussion and warrant attention to prevent potential overtreatment of women with low-risk disease and undertreatment of women at high risk of developing an invasive SBC.^5^ Understanding which women are at risk for developing a subsequent invasive SBC is important to optimally individualize treatment and management.

Racial and ethnic disparities exist among women with DCIS who develop invasive SBC.^6,7,8,9,10^ Native Hawaiian or Other Pacific Islander (NHPI) individuals are 1 of the 5 federally recognized, legally disaggregated US racial categories^11^ and are among the fastest growing populations in the US.^12^ It is becoming increasingly recognized that disaggregating NHPI individuals from other racial and ethnic groups reveals marked health disparities because NHPI individuals have some of the highest cancer mortality rates, the highest chronic disease incidence rates, and the shortest life spans.^11,13,14,15^ Native Hawaiian or Other Pacific Islander women have a higher overall incidence of cancer, including breast cancer, than all Asian American women.^1,15,16,17^ The true cancer incidence among NHPI individuals is reduced by up to half when NHPI individuals are aggregated with Asian American individuals.^1,16,17^ Native Hawaiian women have among the highest rates of breast cancer, with rates nearly 4 times higher than those among Asian women.^16^ Nonetheless, most breast cancer studies continue to aggregate NHPI individuals, and little is known about how NHPI race is associated with prognostication.

Given the importance of optimizing prognostication of women who develop invasive SBC after a DCIS diagnosis, this study aimed to understand the prevalence of invasive SBC among the diverse population in Hawaiʻi and identify risk factors associated with developing invasive ipsilateral SBC (iiSBC) and invasive contralateral SBC (icSBC).

## Methods

### Data Sources and Study Population

Retrospective, deidentified patient data were obtained from the Hawaiʻi Tumor Registry (HTR).^17^ The HTR is 1 of the original 9 National Cancer Institute Surveillance, Epidemiology, and End Results (SEER) registries founded in 1973.^18^ The HTR is responsible for cancer surveillance in the entire state of Hawaiʻi and is a funded registry of the SEER program. Hawaiʻi has the highest proportion in the country of NHPI individuals and individuals of some Asian ethnicities.^19^ Patient data, including demographic, clinical, pathologic, and first-course treatment information, are collected and classified according to uniform national standards.^20,21^ This research was approved as being exempt by the University of Hawaiʻi institutional review board and patient informed consent was waived because the data were deidentified. This study followed the Strengthening the Reporting of Observational Studies in Epidemiology (STROBE) reporting guideline.

The study population was composed of female residents of Hawaiʻi aged 20 years or older who received a diagnosis of a first primary DCIS breast cancer between January 1, 1973, and December 31, 2017. Among these DCIS cases, women with or without a subsequent diagnosis of invasive SBC were evaluated. Women with invasive SBC less than 6 months after their first primary DCIS breast cancer were excluded owing to the possibility of confounding residual or recurrent disease. Patient demographic, clinical, pathologic, and treatment characteristics were evaluated based on the initial DCIS breast cancer diagnoses. Invasive ipsilateral SBC and icSBC were defined by laterality (left vs right) of the first DCIS compared with the invasive SBC, including all quadrants of the disease. Clinical and pathologic characteristics included grade, histologic characteristics, hormone status, and laterality. First course of therapy was classified as breast-conserving surgery (BCS) alone, BCS and radiotherapy (RT), BCS and systemic therapy (chemotherapy and/or hormonal therapy) with or without RT, mastectomy only, mastectomy and systemic treatment, or no treatment. Breast cancer subtype information was available for a subset of patients and was based on the expression of estrogen receptor (ER) and/or progesterone receptor (PR). Self-reported racial and ethnic classifications included the 5 major racial and ethnic populations of Hawaiʻi (Native Hawaiian, White, Japanese, Chinese, and Filipino). To preserve anonymity, less-populous racial and ethnic groups were collectively classified as “minority race and ethnicity,” which included individuals self-identifying as Asian ethnic subgroups (eg, Korean, Vietnamese, and Thai), Other Pacific Islander ethnicities, (eg, Samoan, Micronesian, and Tongan), Black, and American Indian and Alaska Native.

### Statistical Analysis

Data analysis was performed from 2020 to 2021. Statistical analyses were conducted with SAS, version 9.4 (SAS Institute Inc). All statistical tests were 2-sided, and *P* < .05 was considered statistically significant. Women with a primary DCIS diagnosis who developed an invasive SBC were compared with those who did not develop an invasive SBC. Two invasive SBC outcomes were separately evaluated: iiSBC and icSBC. The associations of patient and clinical factors with the risk of iiSBC and icSBC were evaluated by unconditional logistic regression to generate odds ratios (ORs) and 95% CIs. Factors (age, race and ethnicity, diagnosis year, DCIS histologic characteristics, laterality, hormone status, and treatment) that were significant on unadjusted analyses using the threshold of *P* < .05 were included in the adjusted models.

## Results

### Patient Demographic Characteristics, Cancer Characteristics, and Patterns of SBC

A total of 6221 women (age range, 20 to ≥80 years) received a diagnosis of DCIS in Hawaiʻi between January 1, 1973, and December 31, 2017; women were predominantly 50 years of age or older (4817 [77%]) and received a diagnosis between 2000 and 2017 (4452 [72%]), with well or moderately differentiated histologic characteristics (2581 [42%]) and noninfiltrating intraductal DCIS (2383 [38%]) (Table 1). The racial and ethnic distribution of patients with DCIS across the state’s most populous groups were 2270 Japanese women (37%), 1411 White women (23%), 840 Filipino women (14%), 821 Native Hawaiian women (13%), and 491 Chinese women (8%). Women of other minority race and ethnicity collectively comprised 6% of cases (n = 388). Data on ER and PR status were missing for 41% of DCIS cases (ER, n = 2546; PR, n = 2576), largely reflecting earlier periods of diagnoses when hormone receptor testing was not widely used. Of those with hormone receptor status information, most were ER positive (3083 [50%]) and PR positive (2782 [45%]). The first course of treatment included mastectomy only (2011 [32%]), BCS and RT (1306 [21%]), BCS and systemic therapy with or without RT (1295 [21%]), mastectomy and systemic treatment (827 [13%]), BSC alone (584 [9%]), and other treatment (74 [1%]). A few patients (124 [2%]) with DCIS had no treatment.

**Table 1.** Characteristics of Women Who Received an Initial Diagnosis of Primary DCIS in Hawaiʻi, 1973-2017

Characteristics, first DCIS	Women, No. (%) (N = 6221)^a^
Age, y	
≥50	4817 (77)
<50	1404 (23)
Race and ethnicity	
White	1411 (23)
Chinese	491 (8)
Filipino	840 (14)
Japanese	2270 (37)
Native Hawaiian	821 (13)
Minority race and ethnicity^b^	388 (6)
Year of diagnosis	
1973-1989	538 (9)
1990-1999	1231 (20)
2000-2009	2317 (37)
2010-2017	2135 (34)
Grade	
Well or moderately differentiated	2581 (42)
Poorly differentiated or undifferentiated	2033 (33)
Unknown	1607 (26)
DCIS histologic characteristics	
Noninfiltrating, intraductal (NOS)	2383 (38)
Cribriform	593 (10)
Duct carcinoma, solid type	432 (7)
Comedocarcinoma	847 (14)
Intraductal or carcinoma in situ	1455 (23)
Other	511 (8)
Laterality	
Right breast	3050 (49)
Left breast	3170 (51)
Unknown	1 (0.02)
ER status	
Positive	3083 (50)
Negative	592 (10)
Unknown	2546 (41)
PR status	
Positive	2782 (45)
Negative	863 (14)
Unknown	2576 (41)
First course of treatment	
BCS alone	584 (9)
BCS and RT	1306 (21)
BCS and systemic treatment with or without RT^c^	1295 (21)
Mastectomy only	2011 (32)
Mastectomy and systemic treatment	827 (13)
No treatment	124 (2)
Other treatment^d^	74 (1)

Abbreviations: BCS, breast-conserving surgery; DCIS, ductal carcinoma in situ; ER, estrogen receptor; NOS, not otherwise specified; PR, progesterone receptor; RT, radiotherapy.

^a^ Column percentage totals may be slightly less than or greater than 100 owing to rounding.

^b^ Asian ethnic subgroups (eg, Korean, Vietnamese, and Thai), Other Pacific Islander ethnicities (eg, Samoan, Micronesian, and Tongan), Black, and American Indian and Alaska Native.

^c^ Systemic treatment includes chemotherapy and/or endocrine therapy.

^d^ Other treatment includes radiotherapy only, surgery (unspecified) with or without other therapy, and systemic treatment with no surgery.

### Invasive SBCs

Of the 6221 patients with DCIS, 5777 (93%) had no subsequent invasive SBC as of the end of the observation period. A total of 444 women (7%) developed an invasive SBC after their initial DCIS diagnosis. Of the 444 women who developed an invasive SBC, 190 developed iiSBC (43%; median time to SBC diagnosis, 7.8 years [range, 0.5-30 years]), and 254 developed icSBC (57%; median time to SBC diagnosis, 5.9 years [range, 0.5-28.8 years]). Ninety patients who developed an invasive SBC less than 6 months after their initial DCIS diagnosis and 202 patients who developed a second DCIS were excluded from the overall analyses.

### Factors Associated With iiSBC

On unadjusted analyses, age, race and ethnicity, year of diagnosis, histologic characteristics, and first course of treatment were each significantly associated with iiSBC and subsequently included in the adjusted analyses (Table 2). On adjusted analysis, women who developed iiSBC were more likely to be younger than 50 years vs 50 years or older (adjusted odds ratio [aOR], 1.49; 95% CI, 1.08-2.06). Compared with White women, an elevated risk for developing an iiSBC was observed for Native Hawaiian women (aOR, 3.28; 95% CI, 2.01-5.35), Filipino women (aOR, 1.94; 95% CI, 1.11-3.42), and Japanese women (aOR, 1.58; 95% CI, 1.01-2.48). Compared with women who received a diagnosis of their first DCIS between 1973 and 1989, women who received a diagnosis of their first DCIS between 2000 and 2009 (aOR, 0.49; 95% CI, 0.26-0.93) or between 2010 and 2017 (aOR, 0.11; 95% CI, 0.05-0.23) were less less likely to develop an iiSBC. Compared with women who were treated with BCS alone, women who underwent any other treatment had a decreased likelihood of developing iiSBC (BCS and RT: aOR, 0.45; 95% CI, 0.27-0.75; BCS and systemic treatment with or without RT: aOR, 0.40; 95% CI, 0.23-0.69; mastectomy only: aOR, 0.23; 95% CI, 0.13-0.39; mastectomy and systemic treatment: aOR, 0.57; 95% CI, 0.33-0.96). Women receiving no treatment had an increased risk of iiSBC (aOR, 2.29; 95% CI, 1.09-4.80) compared with those receiving BCS alone.

**Table 2.** Risk of iiSBC After a First DCIS Diagnosis^a^

Characteristics, first DCIS	Women, No. (%)^b^		Odds ratio (95% CI)	
	No SBC (n = 5777)	iiSBC (n = 190)	Unadjusted	Adjusted^c^
Age, y				
≥50	4496 (78)	129 (68)	1 [Reference]	1 [Reference]
<50	1281 (22)	61 (32)	1.66 (1.22-2.26)	1.49 (1.08-2.06)
Race and ethnicity				
White	1333 (23)	29 (15)	1 [Reference]	1 [Reference]
Chinese	467 (8)	10 (5)	0.98 (0.48-2.04)	1.09 (0.52-2.28)
Filipino	777 (13)	24 (13)	1.42 (0.82-2.46)	1.94 (1.11-3.42)
Japanese	2094 (36)	71 (37)	1.56 (1.01-2.41)	1.58 (1.01-2.48)
Native Hawaiian	736 (13)	45 (24)	2.81 (1.75-4.52)	3.28 (2.01-5.35)
Minority race and ethnicity^d^	370 (6)	11 (6)	1.37 (0.68-2.76)	1.63 (0.79-3.36)
Year of diagnosis				
1973-1989	485 (8)	19 (10)	1 [Reference]	1 [Reference]
1990-1999	1075 (19)	73 (38)	1.73 (1.04-2.90)	1.02 (0.58-1.78)
2000-2009	2131 (37)	81 (43)	0.97 (0.58-1.62)	0.49 (0.26-0.93)
2010-2017	2086 (36)	17 (9)	0.21 (0.11-0.40)	0.11 (0.05-0.23)
Grade^e^				
Well or moderately differentiated, No./total No. (%)	2424/4341 (56)	71/119 (60)	1 [Reference]	NA
Poorly differentiated or undifferentiated, No./total No. (%)	1917/4341 (44)	48/119 (40)	0.85 (0.59-1.24)	NA
DCIS histologic characteristics				
Noninfiltrating, intraductal (NOS)	2158 (37)	101 (53)	1 [Reference]	1 [Reference]
Cribriform	555 (10)	15 (8)	0.58 (0.33-1.00)	1.01 (0.55-1.84)
Duct carcinoma, solid type	410 (7)	10 (5)	0.52 (0.27-1.01)	1.30 (0.63-2.67)
Comedocarcinoma	807 (14)	19 (10)	0.50 (0.31-0.83)	0.75 (0.45-1.25)
Intraductal or carcinoma in situ	1386 (24)	20 (11)	0.31 (0.19-0.50)	0.74 (0.42-1.28)
Other	461 (8)	25 (13)	1.16 (0.74-1.82)	1.42 (0.89-2.27)
Laterality, No./total No. (%)^e^				
Right breast	2848/5776 (49)	84/190 (44)	1 [Reference]	NA
Left breast	2928/5776 (51)	106/190 (56)	0.82 (0.61-1.09)	NA
ER status, No./total No. (%)^e^				
Positive	2915/3472 (84)	65/77 (84)	1 [Reference]	NA
Negative	557/3472 (16)	12/77 (16)	0.96 (0.52-1.79)	NA
PR status, No./total No. (%)^e^				
Positive	2635/3447 (76)	57/75 (76)	1 [Reference]	NA
Negative	812/3447 (24)	18/75 (24)	1.02 (0.60-1.75)	NA
First course of treatment				
BCS alone	534 (9)	30 (16)	1 [Reference]	1 [Reference]
BCS and RT	1220 (21)	34 (18)	0.50 (0.30-0.82)	0.45 (0.27-0.75)
BCS and systemic treatment with or without RT^f^	1240 (22)	25 (13)	0.36 (0.21-0.62)	0.40 (0.23-0.69)
Mastectomy only	1869 (32)	42 (22)	0.40 (0.25-0.65)	0.23 (0.13-0.39)
Mastectomy and systemic treatment	736 (13)	47 (25)	1.14 (0.71-1.82)	0.57 (0.33-0.96)
No treatment	106 (2)	12 (6)	2.02 (1.00-4.06)	2.29 (1.09-4.80)
Other treatment^g^	72 (1)	0	No estimates^h^	NA

Abbreviations: BCS, breast-conserving surgery; DCIS, ductal carcinoma in situ; ER, estrogen receptor; iiSBC, invasive ipsilateral SBC; NA, not applicable; NOS, not otherwise specified; PR, progesterone receptor; RT, radiotherapy; SBC, second breast cancer.

^a^ Excludes invasive breast cancers diagnosed less than 6 months after DCIS diagnosis.

^b^ Column percentage totals may be slightly less than or greater than 100 owing to rounding.

^c^ Adjusted for variables significant in univariate analysis: age, race and ethnicity, diagnosis year, histologic characteristics, and first course of treatment.

^d^ Asian ethnic subgroups (eg, Korean, Vietnamese, and Thai), Other Pacific Islander ethnicities (eg, Samoan, Micronesian, and Tongan), Black, and American Indian and Alaska Native.

^e^ Missing data: grade (n = 1607), laterality (n = 1), ER (n = 2305), and PR (n = 2330).

^f^ Systemic treatment includes chemotherapy and/or endocrine therapy.

^g^ Other treatment includes radiotherapy only, surgery (unspecified) with or without other therapy, and systemic treatment with no surgery.

^h^ No estimates yielded owing to zero cell values.

### Factors Associated With icSBC

On unadjusted analyses, race and ethnicity, year of diagnosis, and histologic characteristics were each significantly associated with icSBC and subsequently included on adjusted analysis (Table 3). On adjusted analysis, an elevated risk for developing an icSBC was observed for Native Hawaiian women (aOR, 1.69; 95% CI, 1.10-2.61) and Filipino women (aOR, 1.70; 95% CI, 1.10-2.63) relative to White women. Compared with women who received a diagnosis of their first DCIS between 1973 and 1989, women who received a diagnosis of their first DCIS between 2000 and 2009 (aOR, 0.57; 95% CI, 0.36-0.91) or between 2010 and 2017 (aOR, 0.17; 95% CI, 0.09-0.30) were less less likely to develop an icSBC.

**Table 3.** Risk of icSBC After a First DCIS Diagnosis^a^

Characteristics, first DCIS	Women, No. (%)^b^		Odds ratio (95% CI)	
	No SBC (n = 5777)	icSBC (n = 254)	Unadjusted	Adjusted^c^
Age, y				
≥50	4496 (78)	192 (76)	1 [Reference]	NA
<50	1281 (22)	62 (24)	1.13 (0.84-1.52)	NA
Race and ethnicity				
White	1333 (23)	49 (19)	1 [Reference]	1 [Reference]
Chinese	467 (8)	14 (6)	0.82 (0.45-1.49)	0.82 (0.45-1.50)
Filipino	777 (13)	39 (15)	1.37 (0.89-2.10)	1.70 (1.10-2.63)
Japanese	2094 (36)	105 (41)	1.36 (0.97-1.93)	1.30 (0.92-1.84)
Native Hawaiian	736 (13)	40 (16)	1.48 (0.96-2.27)	1.69 (1.10-2.61)
Minority race and ethnicity^d^	370 (6)	7 (3)	0.52 (0.23-1.15)	0.63 (0.28-1.40)
Year of diagnosis				
1973-1989	485 (8)	34 (13)	1 [Reference]	1 [Reference]
1990-1999	1075 (19)	83 (33)	1.10 (0.73-1.67)	1.06 (0.70-1.62)
2000-2009	2131 (37)	105 (41)	0.70 (0.47-1.05)	0.57 (0.36-0.91)
2010-2017	2086 (36)	32 (13)	0.22 (0.13-0.36)	0.17 (0.09-0.30)
Grade^e^				
Well or moderately differentiated, No./total No. (%)	2424/4341 (56)	86/154 (56)	1 [Reference]	NA
Poorly differentiated or undifferentiated, No./total No. (%)	1917/4341 (44)	68/154 (44)	1.00 (0.72-1.38)	NA
DCIS histologic characteristics				
Noninfiltrating, intraductal (NOS)	2158 (37)	124 (49)	1 [Reference]	1 [Reference]
Cribriform	555 (10)	23 (9)	0.72 (0.46-1.14)	1.37 (0.83-2.28)
Duct carcinoma, solid type	410 (7)	12 (5)	0.51 (0.28-0.93)	1.25 (0.65-2.42)
Comedocarcinoma	807 (14)	21 (8)	0.45 (0.28-0.72)	0.69 (0.43-1.12)
Intraductal or carcinoma in situ	1386 (24)	49 (19)	0.62 (0.44-0.86)	1.42 (0.93-2.17)
Other	461 (8)	25 (10)	0.94 (0.61-1.47)	1.22 (0.78-1.92)
Laterality, No./total No. (%)^e^				
Right breast	2848/5776 (49)	118/254 (46)	1 [Reference]	NA
Left breast	2928/5776 (51)	136/254 (54)	0.89 (0.69-1.15)	NA
ER status, No./total No. (%)^e^				
Positive	2915/3472 (84)	103/126 (82)	1 [Reference]	NA
Negative	557/3472 (16)	23/126 (18)	1.16 (0.74-1.85)	NA
PR status, No./total No. (%)^e^				
Positive	2635/3447 (76)	90/123 (73)	1 [Reference]	NA
Negative	812/3447 (24)	33/123 (27)	1.19 (0.79-1.79)	NA
First course of treatment				
BCS only	534 (9)	20 (8)	1 [Reference]	NA
BCS and RT	1220 (21)	52 (21)	1.14 (0.67-1.93)	NA
BCS and systemic treatment with or without RT^f^	1240 (22)	30 (12)	0.65 (0.36-1.15)	NA
Mastectomy only	1869 (32)	100 (39)	1.43 (0.88-2.33)	NA
Mastectomy and systemic treatment	736 (13)	44 (17)	1.60 (0.93-2.74)	NA
No treatment	106 (2)	6 (2)	1.51 (0.59-3.85)	NA
Other treatment^g^	72 (1)	2 (1)	0.74 (0.17-3.24)	NA

Abbreviations: BCS, breast-conserving surgery; DCIS, ductal carcinoma in situ; ER, estrogen receptor; icSBC, invasive contralateral SBC; NA, not applicable; NOS, not otherwise specified; PR, progesterone receptor; RT, radiotherapy; SBC, second breast cancer.

^a^ Excludes invasive breast cancers diagnosed less than 6 months after DCIS diagnosis.

^b^ Column percentage totals may be slightly less than or greater than 100 owing to rounding.

^c^ Adjusted for variables significant in univariate analysis: race and ethnicity, diagnosis year, and histologic characteristics.

^d^ Asian ethnic subgroups (eg, Korean, Vietnamese, and Thai), Other Pacific Islander ethnicities (eg, Samoan, Micronesian, and Tongan), Black, and American Indian and Alaska Native.

^e^ Missing data: grade (n = 1607), laterality (n = 1), ER (n = 2305), and PR (n = 2330).

^f^ Systemic treatment includes chemotherapy and/or endocrine therapy.

^g^ Other treatment includes radiotherapy only, surgery (unspecified) with or without other therapy, and systemic treatment with no surgery.

## Discussion

In this study, we found that factors not previously reported, to our knowledge, were associated with the development of invasive SBC after an initial DCIS diagnosis. By using a more racially and ethnically diverse patient population compared with previous studies, we unmasked racial and ethnic disparities associated with developing an invasive SBC by disaggregating subpopulations of Asian American individuals and NHPI individuals. Our study found that Native Hawaiian and Filipino women had an increased likelihood of developing both iiSBC and icSBC compared with non-Hispanic White women and that Japanese women and younger women had an increased likelihood of developing iiSBC. Unlike previous studies, the cohort of women assessed here was more racially and ethnically diverse and had a large enough sample size to investigate historically overlooked US subpopulations. The data underscore the importance of disaggregating higher-risk racial and ethnic subpopulations, particularly NHPI individuals who are often aggregated with Asian individuals or even excluded from cancer disparity research.

### Current Prognosticators for SBC

Understanding which women are likely to develop an invasive SBC is vital not only to prognosticate disease outcomes, but also to guide management for patients who have received a diagnosis of a first DCIS breast cancer.^22^ Multiple prognosticators exist to estimate local recurrence for a patient with DCIS. For example, the Van Nuys Prognostic Index (VNPI) uses 3 variables (tumor size, surgical margin, and pathologic characteristics) to categorize patients into low-, intermediate-, and high-risk groups.^23^ The Van Nuys Prognostic Index and subsequent iterations have been useful in identifying women at higher risk of developing SBC to guide decisions, such as the necessity for surgery and/or RT.^22^

Race and ethnicity are important factors in estimating the risk of developing invasive SBC after DCIS. African American women have a high risk of developing invasive SBC and DCIS, both ipsilaterally and contralaterally.^6,7,8,9^ Latina women have increased rates of developing subsequent ipsilateral DCIS.^6^ Thus, there is increasing evidence to suggest that a patient’s race and ethnicity are important prognosticators and may factor into clinical decision-making for patients who have received a diagnosis of an initial DCIS.

Liu and colleagues^6^ assessed 18 National Cancer Institute SEER registries and found that of the 9% of women who developed SBC after an initial noninvasive breast cancer diagnosis (70% of which were invasive SBC), Black and Asian women with DCIS had higher risks of developing biologically aggressive ipsilateral SBC compared with White women. They also reported that ipsilateral SBC occurred more often in Black and Hispanic women compared with White women. In their study, there were no statistical differences in ipsilateral SBC between White women and Asian American and NHPI (AAPI) women, with the AAPI ethnic subanalysis excluding Other Pacific Islander indivduals. This and many studies used the antiquated “AAPI” terminology that combines 2 groups legally disaggregated during the Clinton Administration, ultimately masking true cancer disparities for NHPI women.^11,15^

### Underrepresented US Populations

In this study, we present data from one of the most ethnically unique US populations, with a large enough sample size to investigate smaller subsets of the US population. The state in which these data are derived, Hawaiʻi, has no ethnic majority and has the highest proportion of multiethnic individuals in the country.^24^ The HTR contains the largest number of Native Hawaiian patients and is useful for disaggregating racial and ethnic data for these populations. Thus, the HTR has been curated with granular data to assess racial and ethnic US subpopulations, such as the Native Hawaiian subpopulation, that are underrepresented in other SEER databases.

Despite the existing evidence, it is poorly recognized that when they are properly disaggregated from Asian American individuals, NHPI women have higher morbidity and mortality rates for most cancers, including breast cancer, as well as other chronic diseases, compared with Asian American individuals.^14,15,17^ The continued aggregation of Asian American individuals with NHPI individuals masks existing health disparities and further exacerbates health inequity.^11^ For example, Torre and colleagues^16^ highlight that Native Hawaiian women have nearly a 4-fold higher risk of breast cancer compared with some Asian ethnic subgroups. Cancer mortality among NHPI individuals is not improving, possibly owing to increased late-stage cancers among NHPI individuals.^25^ Despite these high rates, there is minimal representation of NHPI individuals in clinical and genomic studies.^26^

We found that Native Hawaiian women are at the highest risk of developing invasive SBC after a primary DCIS. Compared with White women, Native Hawaiian women had greater odds of developing iiSBC and icSBC. Although consistent with previous data highlighting the increased incidence rates of breast cancer among NHPI individuals,^27^ our study specifically identifies Native Hawaiian race as a significant risk factor associated with the development of iiSBC and icSBC. Understanding the underlying social determinants of health may explain in part why NHPI individuals have such high incidence rates of SBC and primary breast cancer in general. Native Hawaiian or Other Pacific Islander individuals have higher mean body size, greater adult-onset weight gain, and higher rate of tobacco and alcohol abuse, as well as more limited access to health care and poorer neighborhood socioeconomic status.^15,28,29,30,31^ Understanding these social determinants of health underscores the importance of proper disaggregation of Asian American and NHPI individuals.

In addition to Native Hawaiian women, our data also reveal that there are Asian subpopulations that disproportionately experience invasive SBC. Filipino women had a significantly higher likelihood of developing both iiSBC and icSBC compared with White women. Japanese women were also shown in this study to have an increased risk of developing an iiSBC. These data contrast with previous studies that have shown no increased risk of iiSBC in Asian ethnic subpopulations compared with White women.^6^ These data highlight that there may be unappreciated heterogeneity within Asian subpopulations, which has been suggested previously.^14^ It may be important to assess in future studies whether there are alternative categories other than a single Asian group (eg, Southeast Asian, East Asian, and South Asian).

### Additional Factors: Age, Time, Treatment, and Laterality

Beyond race and ethnicity, previous studies have demonstrated other factors associated with an increased risk of developing SBC. We found that women younger than 50 years of age had greater odds of developing iiSBC compared with women 50 years of age or older. This association is consistent with a previous study that found that not only were younger women at an increased risk of developing iiSBCs but also had an associated increased mortality rate and more biologically aggressive tumor characteristics.^32^ Younger women are typically healthier, with fewer comorbid conditions, and have longer life expectancies agnostic to their cancer diagnosis and thus reasonably have more life span to develop SBC. This is critical given that younger women with breast cancer have an associated 12-fold increased risk of death compared with the general population.^33^

The time of initial DCIS diagnosis has multiple implications for the development of a subsequent SBC. We found that, after adjusting for confounders, the year of initial DCIS diagnosis was associated with the development of invasive SBCs. We found that, compared with the earlier time points (1973-1989), women who received a diagnosis of breast cancer after 2000 had reduced odds of developing an invasive SBC. This finding may be due to the increasing prevalence of mammography screening between 1987 and 2000; screening rates increased from 29% to 70% during this period.^34^ The ability to detect and localize smaller noninvasive tumors earlier may have affected clinical outcomes. Our observation of decreased odds of SBC in the 2010s compared with earlier decades is consistent with previous reports using national data.^10^ Decreases in SBC risk in the later decades may also be explained in part by the development of new therapeutic strategies, including lumpectomy with adjuvant RT and endocrine therapy.^35,36,37,38^

Treatment of DCIS includes mastectomy, BCS or lumpectomy, endocrine therapy, and RT.^5^ Compared with BCS alone, RT in combination with BCS significantly reduces the chance of local recurrence^39^ and may reduce overall breast cancer mortality.^38^ Breast-conserving surgery alone has been shown to be associated with a 10-year local recurrence rate of 20% to 44%. Modern treatment augments BCS with either RT (50% reduction of local recurrence) and/or endocrine therapy (37% relative risk reduction of developing ipsilateral and contralateral SBC).^3,39^ Although, in our study, we focused on the development of SBC rather than on local recurrence, for which data are not collected by SEER registries, we found that, compared with patients who underwent BCS alone, the odds of developing an iiSBC were reduced by approximately half in patients who received RT after BCS. We found that this was also true for patients who underwent mastectomy. In contrast, women who underwent no treatment had roughly double the odds of developing an iiSBC. This finding is also consistent with large national meta-analyses, suggesting the applicability of this database to national-level data.^3^

Differences between the factors associated with iiSBC and the factors associated with icSBC have been previously investigated in the context of race and ethnicity. A previous report suggested a greater incidence of iiSBC among Black and Hispanic women but no difference in the aggregate Asian American and NHPI group.^6^ In that study, like many others, the data were inappropriately aggregated into a single AAPI group. Thus, given that our data appropriately disaggregated NHPI individuals, we were able to investigate breast cancer disparities among NHPI individuals that have not been previously appreciated. As for icSBC, our data are consistent with a previous report suggesting that Asian American and NHPI individuals have higher rates of contralateral breast tumors.^6^ Given our ethnically disaggregated findings, it may be the case that, in previous studies, some Asian subsets (eg, Filipino women) had disproportionately increased odds of developing icSBC, increasing the odds within the aggregate AAPI category.

### Limitations

There are multiple limitations to this study. The generalizability of the study is important to take into consideration. The women in this study were all residents of Hawaiʻi and thus may not be representative of the entire US population. However, the comparison of Native Hawaiian women vs non-Hispanic White women still provides insight. This is especially true given that the National Cancer Institute SEER database used in this study has the largest number and the largest proportion of Native Hawaiian individuals represented in the data set. Although we focused on the 5 most represented racial and ethnic groups in the sample, Black, American Indian and Alaska Native, and Other Pacific Islander individuals were not represented with sufficient sample sizes for a meaningful statistical analysis. Subset data for groups with small numbers could not be shown to protect patient confidentiality. The lack of association with SBC risk observed for certain variables (ER status, PR status, and tumor grade) may have been influenced by their large proportion of missing values.

## Conclusions

This cohort study suggests that Native Hawaiian women, Filipino women, and women who received a diagnosis of DCIS before 2000 have higher odds of developing both iiSBC and icSBC. It also suggests that women younger than 50 years and Japanese women have an increased risk for developing iiSBC. This study highlights racial and ethnic disparities in the risk of developing invasive SBC that were not, to our knowledge, previously appreciated among Native Hawaiian women and subpopulations of Asian American women compared with White women. This finding may help oncologists understand the association of race and ethnicity with the risk of developing invasive SBC in these understudied populations.
